# Insights into orbital morphological features and fracture patterns in medial and inferior wall fracture: a retrospective cohort study

**DOI:** 10.1038/s41598-023-47941-9

**Published:** 2023-11-27

**Authors:** Junjie Yang, Yali Du, Zhengyun Zhen, Shuang Wang, Sumei Zhao, Guang Zhao, Bingjie Shi

**Affiliations:** 1 Department of Ophthalmology, Union Hospital, Tong Medical College, Huazhong University of Science and Technology, Wuhan, China; 2 Hubei Hospital of Integrated Traditional Chinese and Western Medicine, Wuhan, China; 3 Hebei General Hospital for Veterans, Xingtai, China; 4 Tongji Medical College, Huazhong University of Science and Technology, Wuhan, China

**Keywords:** Eye diseases, Anatomy, Medical research, Signs and symptoms

## Abstract

This study investigated the orbital morphological features that lead to fractures at different sites by comparing patients with isolated inferior wall fracture (IWF) to patients with isolated medial wall fracture (MWF). This study analyzed the orbital morphologic characteristics of all orbital fracture patients who underwent orbital computed tomography (CT) scans between January 2017 and October 2022. On CT scans, the bony structures of the orbit were measured. We investigated the bilateral symmetry of orbital. In addition, orbital morphological differences were compared between patients with fractures of the medial wall and those with fractures of the inferior wall. A total of 135 patients with orbital fractures were included in the study. Of these, 91 were isolated MWFs and 44 were isolated IWF. We confirmed the symmetry of bilateral orbits and measured the orbit of the uninjured side. No differences were found between the MWF group and the IWF group in terms of ocular prominence, horizontal orbital diameter, orbital rim angle, sagittal orbital depth, sagittal orbital depth, and angle of inferior wall inclination. The distance between the infraorbital nerve (ION) entry point and the orbital rim was significantly smaller in the inferior lateral wall fracture group than in the MWF group (11.87 ± 2.54 vs 14.90 ± 4.64, P < 0.001), and the percentage of type 1 ION was significantly lower in the IWF group than in the MWF group (40.9% vs 65.9%, P = 0.012). We demonstrated the symmetry of bilateral orbits and found that when the point where the ION enters the infraorbital canal is near the orbital rim, patients are more prone to suffering a fracture of the inferior wall after orbital trauma. It is less likely for patients with type 1 ION to suffer an IWF following an orbital fracture.

## Introduction

Orbital fracture is a common traumatic condition of the eye. It can result from impacts during ball sports, boxing, or object hitting. About 57% of all maxillofacial fractures are orbital fractures^1^. It is possible for an orbital fracture to occur as a consequence of the sudden posterior displacement of the eye following impact and an increase in intraorbital pressure (hydraulic theory) or due to a direct transfer of force to the anterior orbit following an impact on the orbital rim (flexion phenomenon)^2^. Orbital walls are particularly weak in the medial and inferior areas. Since the medial wall is composed of a thin bony structure, it is also referred to as "cardboard." In the inferior wall, there are areas of weakness due to the penetration of the infraorbital nerve (ION). Due to this, both the inferior and medial walls are susceptible to fracture when external forces are applied.

In our clinic, we have observed that some patients with mild traumatic impact have isolated fractures of the medial wall, and others have isolated fractures of the inferior wall. According to Park et al., patients with steeper and more convex orbital floors are more likely to sustain isolated inferior wall fracture (IWF). However, the results of the study did not show a significant difference^3^. The study by Won Kyung Song et al. found that patients with fewer ethmoid air cell septa and a larger lamina papyracea area per septum were more likely to suffer a medial wall fracture (WMF)^4^. Nevertheless, this study only examined the impact of septal sinus structure on MWF, which is not sufficient to answer our questions.

The ION is a peripheral branch of the maxillary division of the trigeminal nerve. The maxillary nerve traverses the cranial base through the foramen rotundum and gains access to the orbit via the inferior orbital fissure. Typically, it courses anteriorly within a canal or groove in the orbital floor, assuming the identity of the ION, and subsequently exits through the infraorbital foramen (IOF) into the facial soft tissues. Elisabeth H classified the ION into three types by observing the descent of ION within the maxillary sinus and changes in the position of the IOF in 1000 patients' computed tomography (CT) scans. These observations may assist surgeons in preventing iatrogenic ION injury^5^. This study has also inspired us, suggesting that different classifications and morphologies of ION may alter the stability of the inferior orbital wall, ultimately influencing the outcome of trauma.

Therefore, we conducted this study and collected orbital CT data from patients with isolated MWF and isolated IWF for study to explore the effect of orbital morphologic features on the site of orbital fractures.

## Materials and methods

### Study population and study design

**Figure 1:**
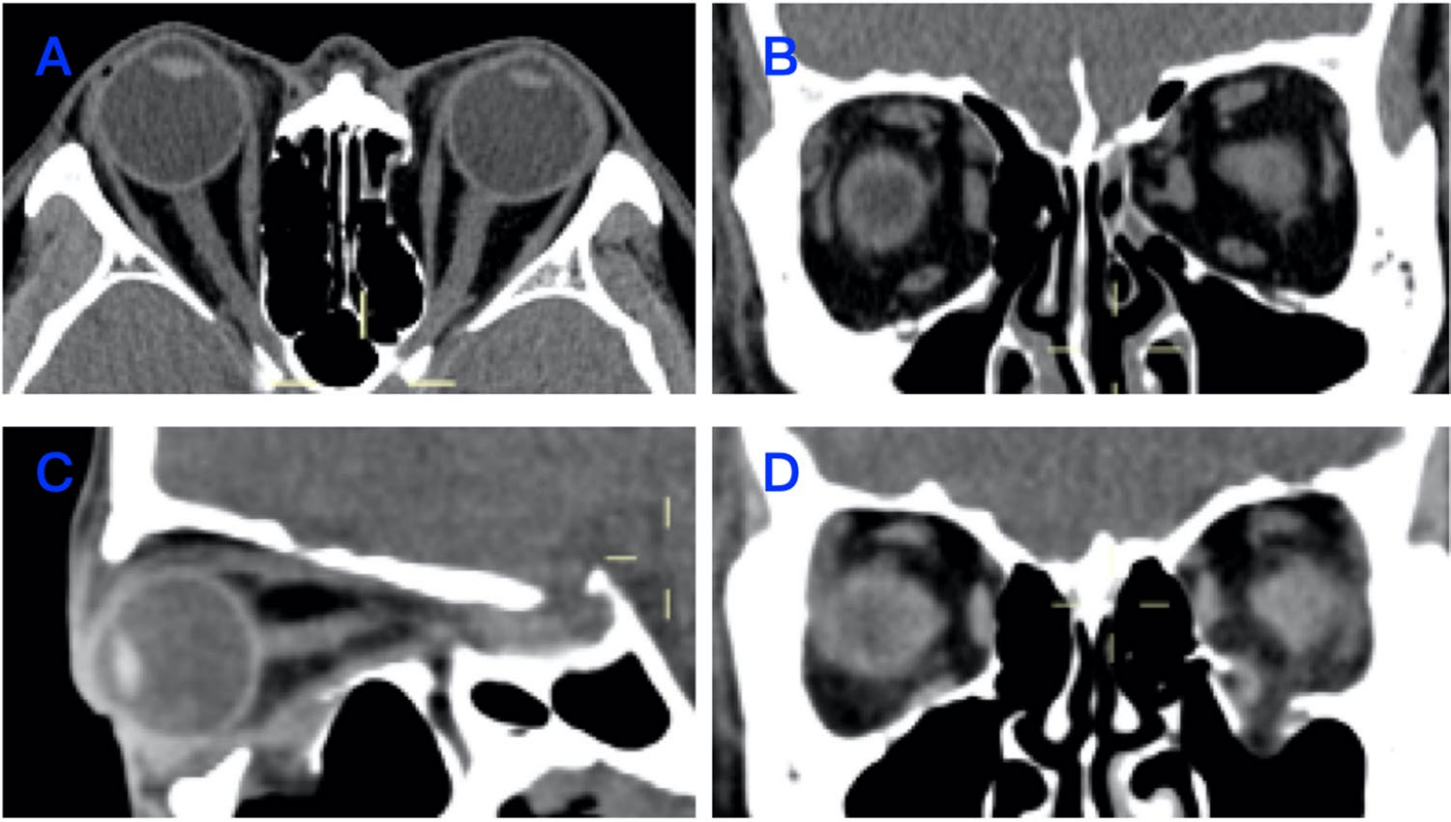
Examples of medial wall and inferior wall orbital fractures. (**A**) Axial orbital CT for MWF. (**B**) Coronal orbital CT for MWF. (**C**) Sagittal orbital CT for IWF. (**D**) Coronal orbital CT for IWF.

This was a retrospective cross-sectional study. We collected information from 135 patients who received treatment for orbital fractures between January 1, 2017, and October 1, 2022. These patients were treated at the Ophthalmic Department of the Union Hospital of Tongji Medical College, Huazhong University of Science and Technology, China. We divided the patients into two groups based on the type of fracture: patients with isolated IWF and patients with isolated MWF (Fig. 1).

Inclusion criteria were as follows: (a) Patients ≥ 18 years of age; (b) Single eye injury with an isolated MWF or IWF; (c) No previous orbital trauma or surgery for both eyes; (d) CT of the orbit following the injury without treatment prior to the orbital CT; (e) Have no disease related to orbital developmental malformations.

Exclusion criteria were as follows: (a) Age < 18 years; (b) A history of previous ocular trauma or surgery on the injured eye; (c) A history of ocular trauma or orbital surgery on the contralateral eye; (d) Diagnosed with an orbital developmental malformation-related disease; (e) Bilateral orbital fractures or fractures involving more than one orbital wall; (f) Orbital fractures extending to the orbital rim; (g) Insufficient CT data or CT scans obtained after orbital fracture surgery.

### Data collection

Data collection was conducted by the electronic medical record system of the Union Hospital of Tongji Medical College, Huazhong University of Science and Technology. The CT data were obtained from the picture archiving and communication system. The orbital CT scans were conducted using acquisition parameters of 120 kV (peak), 200 mA, a display field of view of 160 mm, and a pitch ranging from 0.8 to 1.0. We set the level and width of the window to 450/1500 Huntsfield units (HU). For the identification of type 2 ION, the level/width of the window was set to 800/2000 HU. Each parameter was measured by three ophthalmologists separately, and the average of three measurements was taken. The data were collected and managed within the EDC data collection and management system (study.empoweredc.com, Shanghai, China).

**Figure 2:**
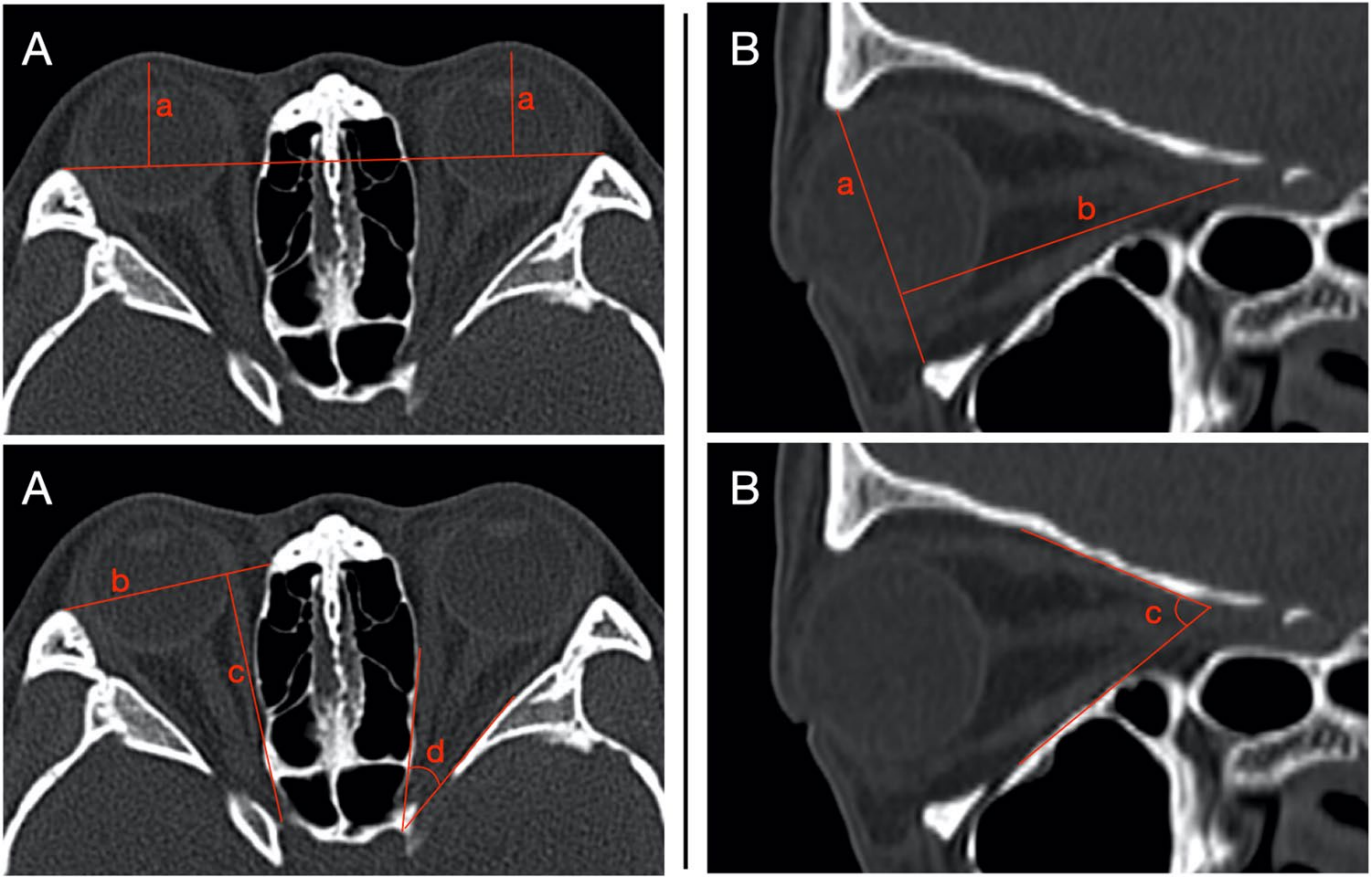
Illustration of orbital parameter measurements. (**A**) On axial CT. (a) Ocular prominence: the vertical distance between the most prominent point of the eye and the interzygomatic line. (b) Horizontal orbital diameter: the distance between the medial and lateral orbital rims. (c) Axial orbital depth: the distance between the apex of the orbit and the interzygomatic line. (d) Orbital rim angle: the angle formed by the medial and lateral walls of the orbit. (**B**) On sagittal CT. (a) Vertical orbital diameter: the distance between the superior and inferior orbital rims. (b) Sagittal orbital depth: the distance from the orbital apex to the orbital orifice. (c) Angle of inferior wall inclination: it is the angle formed between the inferior orbital wall and the sagittal orbital depth line.

**Figure 3:**
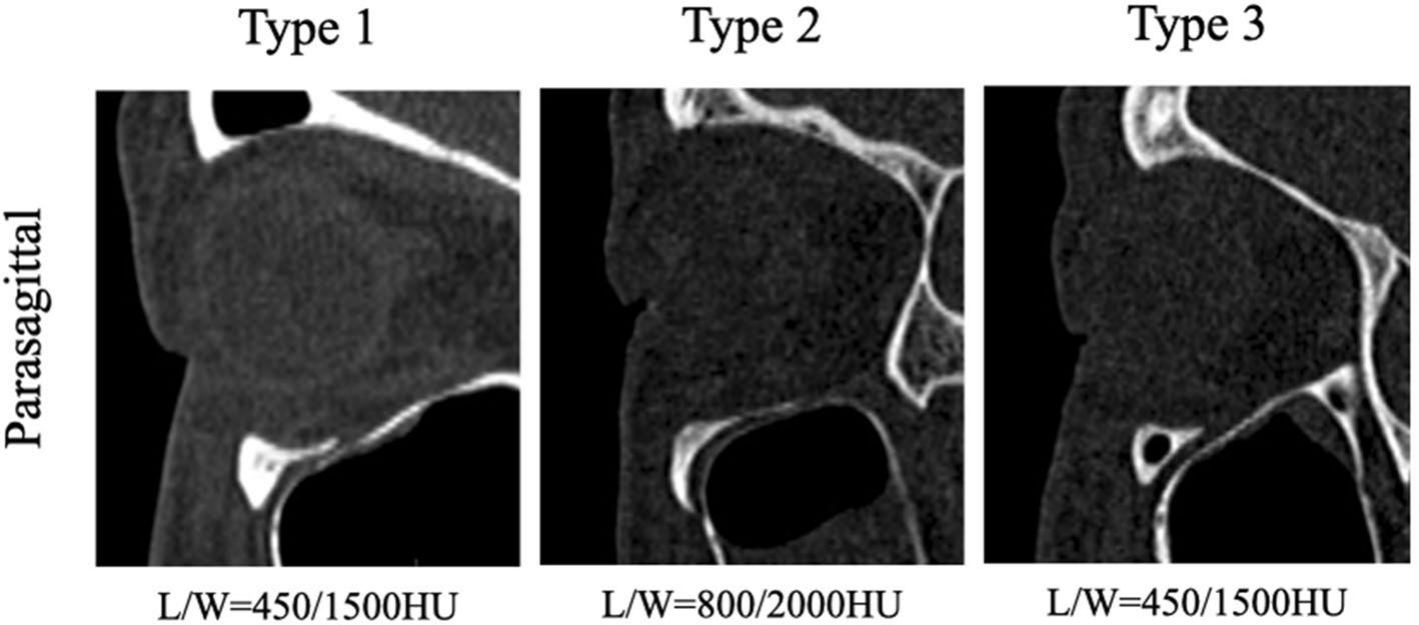
Types of ION. Type 1: the nerve is completely contained within the roof of the sinuses. Type 2: the nerve canal is located beneath the roof, but is contiguous with the roof. Type 3: the nerve descends into the sinus lumen, suspended from the sinus roof within the septum or lamella of the infraorbital ethmoid. L/W is short for window level/width settings.

We measured the ocular prominence, horizontal orbital diameter, axial orbital depth, and orbital rim angle on axial CT (Fig. 2). Measured vertical orbital diameter, sagittal orbital depth, and angle of inferior wall inclination on sagittal CT (Fig. 2). In the sagittal CT, we also categorized the ION into type 1, type 2, and type 3 (Fig. 3), and measured the distance of ION entry point from the orbital rim and the length of the descending portion of the ION.

### Statistical analysis

Statistical analysis of quantitative data involved determining mean, standard deviation (X ± SD), and median (Q1–Q3). Qualitative data were presented using frequencies (%). Kolmogorov–Smirnov tests were conducted to verify normality assumptions.

To detect differences between groups, t-tests and Wilcoxon rank sum tests were used based on data distribution. Chi-square tests were applied for categorical data.

Orbital fracture analysis used contralateral side measurements as proxies. A consistency test ensured bilateral structure consistency. Paired t-tests and chi-square tests were used for continuous and categorical variables, respectively. Scatter plots depicted bilateral parameter differences.

This validation allowed using contralateral data as representative, offering insights into orbital characteristics while minimizing injury-induced biases.

In addition, ∆ represented the parameters of the uninjured orbit minus the parameters of the injured orbit, while Σ represented the parameters of the uninjured orbit plus the parameters of the injured orbit. These calculations enabled the assessment of changes or relationships between the two sets of orbital data.

The statistical analysis was conducted using R version 4.0.3 (https://www.R-project.org) and EasyR (https://www.easyr.cc Solutions, Inc., Shanghai). Statistical significance was determined by P values < 0.05 in all analyses.

### Ethics approval and consent to participate

This study was approved by the Human Ethics Committee of Wuhan Union Hospital, Tongji Medical College, Huazhong University of Science and Technology. The study was conducted in accordance with the Declaration of Helsinki. Due to the retrospective nature of the study and the de-tracking and anonymization of various patient data by the cohort, informed consent was waived by the Human Ethics Committee of Wuhan Union Hospital, Tongji Medical College, Huazhong University of Science and Technology.

## Results

### Patients’ characteristics

A total of 169 patients were enrolled in the study at the beginning. Of these, 20 were adolescents (< 18 years of age), 5 patients had previous ocular trauma, 3 patients had a history of previous orbital surgery, and 6 patients had incomplete CT data, leaving 135 patients with information for the final analysis.

The baseline characteristics of the patients are listed in Table 1. There were 104 males (77%) and 31 females (23%). The mean age of the included patients was 40.17 ± 12.94 years. There were 91 (67.4%) MWF and 44 (32.6%) IWF. As a result of ION typing, we observed 78 cases (57.8%) of type 1, 39 cases (28.9%) of type 2 and 18 cases (13.3%) of type 3.

**Table 1:** Baseline characteristics of patients.

	Mean ± SD
N	135
Age (year)	40.17 ± 12.94
Male (%)	104 (77.0%)
Ocular prominence (mm)	16.90 ± 2.61
Horizontal orbital diameter (mm)	37.24 ± 2.10
Axial orbital depth (mm)	40.41 ± 2.37
Orbital rim angle (°)	45.60 ± 5.12
Vertical orbital diameter (mm)	34.05 ± 2.11
Sagittal orbital depth (mm)	46.55 ± 2.69
Angle of inferior wall inclination (°)	23.46 ± 5.82
Distance of ION entry point from the orbital rim (mm)	13.91 ± 4.29
Length of the descending portion of ION (mm)	14.52 ± 3.85
Anatomical variant of ION typing (%)	N (%)
Type 1	78 (57.8%)
Type 2	39 (28.9%)
Type 3	18 (13.3%)
Fracture site	N (%)
Medial wall (%)	91 (67.4%)
Inferior wall (%)	44 (32.6%)

Data are presented as mean ± standard deviation (SD), or numbers (percentages).

### Bilateral symmetry validation of orbits

**Figure 4:**
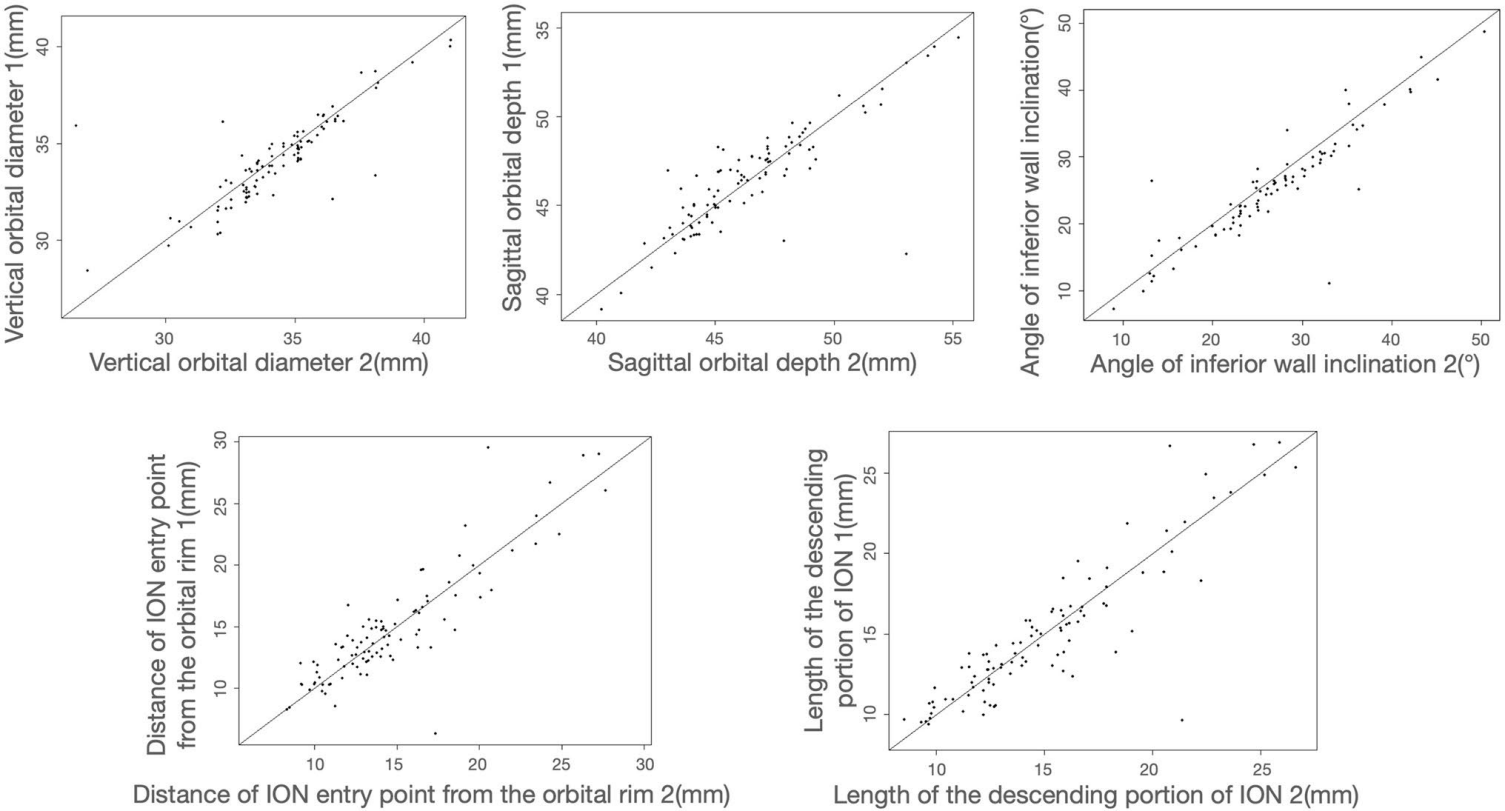
Bilateral orbital symmetry demonstration. The y-axis represents the parameters of the uninjured orbit. The x-axis represents the parameters of the injured orbit.

**Figure 5:**
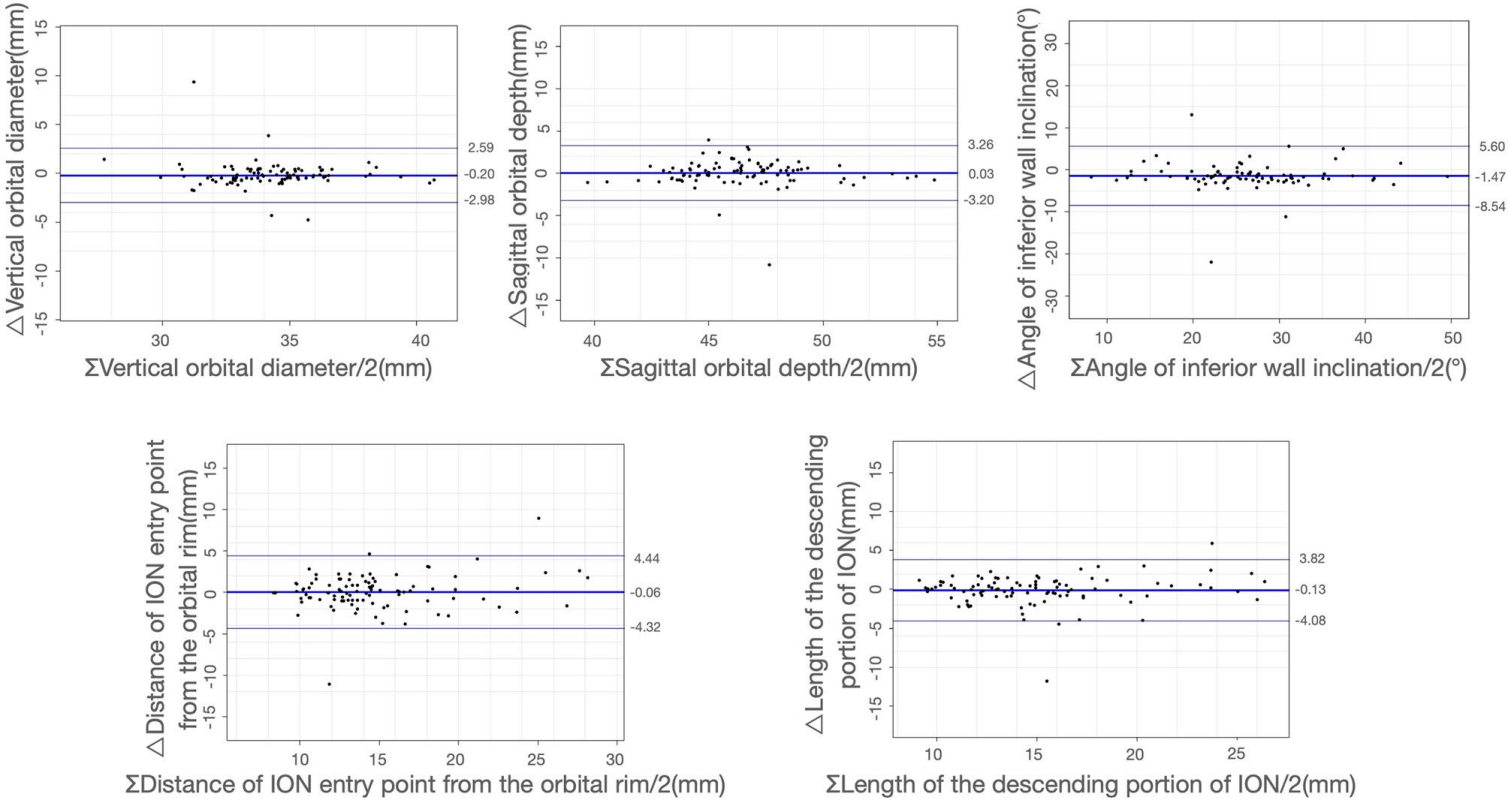
Bilateral orbital symmetry consistency test. △ represents the parameters of the uninjured orbit minus the parameters of the injured orbit, Σ represents the parameters of the uninjured orbit plus the parameters of the injured orbit.

According to Song WK, the medial wall of bilateral orbits is symmetrical^4^. He conducted a study on orbital CT scans of 118 healthy individuals and found that the medial walls of both orbits in the same individual exhibited symmetry. Therefore, we examined orbital symmetry in the sagittal plane. In bilateral orbits of 91 patients with isolate MWF, vertical orbital diameter, sagittal orbital depth, angle of inferior wall inclination, anatomical variation of ION type, distance of ION entry point from the orbital rim, and length of the descending portion of ION were measured. We compared the symmetry of the above data (Fig. 4) and performed a consistency test on the measured parameters of the bilateral orbits of the same patient (Fig. 5). Statistically significant differences were not observed between the above measured parameters of the bilateral orbits (Table 2). The orbital ION typing was the same in 90(98.9%) individuals bilaterally, differing only in 1. Indicating that the bilateral orbits are well symmetrical and that measurements of the uninjured orbit can be used to represent the condition of the injured orbit prior to injury.

**Table 2:** Consistency test on the measured parameters of the bilateral orbits.

	Uninjured orbit	Injured orbit	P-value*
N	91	91	
Horizontal orbital diameter (mm)	34.13 ± 2.22	34.35 ± 2.33	0.3373
Axial orbital depth (mm)	46.46 ± 2.85	46.44 ± 2.91	0.6874
Angle of inferior wall inclination (°)	26.02 ± 7.41	27.55 ± 7.34	0.0972
Distance of ION entry point from the orbital rim (mm)	14.86 ± 4.52	14.70 ± 4.34	0.812
Length of the descending portion of ION (mm)	15.06 ± 4.28	15.20 ± 4.00	0.6978
Anatomical variant of ION typing (%)			
Type 1	60 (65.93%)	61 (67.03%)	0.9849
Type 2	23 (25.27%)	22 (24.18%)	
Type 3	8 (8.79%)	8 (8.79%)	

Data are presented as mean ± standard deviation (SD), or numbers (percentages).

*Paired t-test or Paired Chi-square test.

### Comparison of orbital morphological variability between the two groups

Upon comparing the MWF group with the IWF group (Table 3), there were no significant discrepancies observed in gender distribution (p = 0.542). However, substantial variances in age were evident, indicating a notably higher average age in the MWF group compared to the IWF group (p < 0.001). The mean distance between the ION entry point and the orbital rim was 14.90 ± 4.64 in the MWF group and 11.87 ± 2.54 in the IWF group, with a significant difference (P < 0.001). The data indicate that patients whose ION entry points are close to the orbital rim are more likely to suffer IWF after experiencing orbital trauma. There were 60 (65.9%) type 1 ION in the MWF group and 18 (40.9%) type 1 ION in the IWF group. The proportion of type 1 ION was significantly higher in the MWF group than in the IWF group (P = 0.012), indicating that for patients with type 1 ION, they were less likely to develop IWF after suffering orbital trauma. We noted some difference in axial orbital depth between the two groups as well, (40.09 ± 2.50 vs 41.08 ± 1.95, P = 0.029). However, the difference was rather small, and no significant difference was seen in sagittal orbital depth (46.46 ± 2.85 vs 46.73 ± 2.36, P = 0.394), thus we did not pay attention to this data.

**Table 3:** Comparison of bony orbit in two groups.

	Medial wall fracture group	Inferior wall fracture group	P-value
N	91	44	
Sex			
Male	72 (79.1%)	32 (72.7%)	0.542
Female	19 (20.9%)	22 (27.3%)	
Age (year)			
< 30	17 (18.7%)	19 (43.2%)	< 0.001*
30–40	21 (23.1%)	15 (34.1%)	
40–50	21 (23.1%)	7 (15.9%)	
≥ 50	32 (35.2%)	3 (6.8%)	
Ocular prominence(mm)	16.75 ± 2.46	17.21 ± 2.91	0.310
Horizontal orbital diameter(mm)	37.01 ± 2.15	37.69 ± 1.94	0.060
Axial orbital depth(mm)	40.09 ± 2.50	41.08 ± 1.95	0.029*
Orbital rim angle(°)	45.47 ± 5.02	45.86 ± 5.38	0.818
Vertical orbital diameter(mm)	34.13 ± 2.22	33.87 ± 1.88	0.487
Sagittal orbital depth(mm)	46.46 ± 2.85	46.73 ± 2.36	0.394
Angle of inferior wall inclination(°)	23.11 ± 5.64	23.36 ± 6.24	0.761
Distance of ION entry point from the orbital rim(mm)	14.90 ± 4.64	11.87 ± 2.54	< 0.001*
Length of the descending portion of ION(mm)	15.06 ± 4.28	13.37 ± 2.42	0.0461
Anatomical variant of ION typing (%)			
Type 1	60 (65.9%)	18 (40.9%)	0.012*
Type 2	23 (25.3%)	16 (36.4%)	
Type 3	8 (8.8%)	10 (22.7%)	

Data are presented as mean ± standard deviation (SD), or numbers (percentages), *P < 0.05.

## Discussion

The purpose of this study was to investigate the differences in orbital bony parameters between patients with fractures at different sites of the orbit. The orbital morphology of patients with isolated IWF differed from those with isolated MWF. There was a significant difference in the distance of the ION entry point from the orbital rim, as well as a significant difference in the type of ION between the two groups. In the IWF group, the mean ION entry distance was shorter than in the MWF group (11.87 ± 2.54 vs14.90 ± 4.64, P < 0.001). In the MWF group, the percentage of type 1 ION was significantly higher than in the IWF group (65.9% vs 40.9%, P = 0.012). It is suggested that patients having a shorter ION entry point distance from the orbital rim are more likely to have IWF after trauma, and patients with type I ION are less likely to have IWF after trauma.

A total of 135 participants were included in our study, 31 (23%) female and 104 (77.0%) male. It is similar to Thomas's findings^6^. MWF accounted for 91 (67.4%) cases and IWF accounted for 44 (32.6%) cases. There were more MWF than IWF. It is similar to the studies by Michelle, Khojastepour and Choi^7–9^. However, Jung EH reported more IWF (71.5%) than MWF (28.5%) in 3415 Korean patients^10^. The bones in the medial and inferior walls of the orbit are the thinnest, resulting in poor strain resistance, making them more susceptible to fracture. There has been evidence that ethnic groups differ in their vulnerability to orbital fractures. Asians and Afro-Caribbeans are more likely to suffer a fracture to the medial wall, while Caucasians are more likely to suffer a fracture to the orbital floor^11–13^. This study, which included all Chinese individuals, also found a higher percentage of MWF.

### The same patient has bilateral orbital symmetry

As the orbital structures on the traumatized side had been destroyed, we analyzed the uninjured side of the orbit. According to Song WK, who investigated the issue of orbital symmetry in 118 CTs of orbits obtained from a healthy population, both orbital walls had good symmetry^4^. Takahashi et al. observed good symmetry in the bilateral orbits of 22 cadavers^14^. We compared the bilateral sagittal orbital bony parameters, including vertical orbital diameter, sagittal orbital depth, inferior wall declination angle, typing of the ION, distance of the ION entry point from the orbital rim, and length of the descending portion of the ION in patients with MWF, and found no significant differences. The findings indicate that the same patient has bilateral orbital symmetry and that the uninjured orbit can be used to represent the parameters of the injured orbit prior to injury.

### The distance from the orbital rim to the ION entry point differs between the IWF group and the MWF group

It is common for orbital floor fractures to extend into the infraorbital sulcus as well as the infraorbital fissure^15^. In the anterior part of the orbit, the ION is located in a canal known as the infraorbital canal. In the middle and posterior regions of the orbit, it is located in a groove known as the infraorbital sulcus. As the infraorbital sulcus connects with the infraorbital canal, the orbital floor is at its weakest point^16, 17^. Here, the very thin bone offers the least resistance to the strength of the orbital bone. We refer to this point as the ION entry point and have found that for patients with IWF, the distance from the orbital rim to the ION entry point is significantly shorter than for those with MWF. In a study of 10 orbits obtained from five cadavers, Sagar Patel et al. found that small impact forces may be sufficient to cause anterior fractures to the inferior orbital wall. The hydraulic theory of intraorbital pressure transmission may explain this phenomenon^18^. Whenever the ION entry point is closer to the orbital rim, the weak area of the infraorbital wall is closer to the front. In the event of a pressure change, the weak bone wall is more likely to fracture.

### Patients with type 1 ION are less likely to have IWF

The ION leaves the orbital floor through the infraorbital canal and penetrates through the IOF in the anterior maxilla. In the population, the morphology of the ION differs. According to Fersions et al., ION is classified into three types based on its anatomical variants. Type 1: the nerve is completely contained within the roof of the sinuses. Type 2: the nerve canal is located beneath the roof, but is contiguous with the roof. Type 3: the nerve descends into the sinus lumen, suspended from the sinus roof within the septum or lamella of the infraorbital ethmoid^5^. Fersions' study found 12.5% of patients with type 3 ION. Junhyung Kim’s study reported 10.5% of type 3 ION^19^. Lantos et al. studied 500 orbital CTs and found 10.8% of type 3 ION^20^. The results of our study indicate that 13.3% of the individuals had type 3 ION, which is consistent with previous research. In comparison to patients with MWF (40.9%), the percentage of type 1 ION was significantly lower in patients with IWF (65.9%). Type 1 ION have the strongest infraorbital canal of all three types. The distance between the inferior orbital rim and the superior edge of the inferior orbital foramen is significantly shorter in type 1 than in type 2 and type 3. The descending portion of the ION is usually shorter as well. It can be less lax in the case of orbital floor fractures. Thus, the inferior displacement of fractured orbital bone and soft tissue can be less severe in patients with type 1 ION than in patients with other types of ION^19^. This may reflect the fact that patients with type 1 ION are less likely to have IWF.

### Novelty and contribution to existing literature

We emphasize the originality of our study, which explored the differences in orbital skeletal morphology between IWF and MWF patients. Amidst the limited presence of similar investigations within the existing literature, our research uniquely delved into the potential impact of different classifications and morphologies of ION on the site of fractures. The study deepens the understanding of the process of orbital fractures occurring after trauma for clinical practitioners. By providing a fresh perspective, our study enriches the scientific discourse in this field and may offer clinicians a deeper understanding of the condition.

### Advantages and limitations

The main advantages of this study are as follows: CT measurements were used to obtain data for orbits, ensuring the accuracy of orbital parameter measurements. Despite being an observational study, which is inevitably subject to confounding factors, we rigorously adjusted for these factors and assessed their robustness through sensitivity analysis.

The limitations of this study are as follows: the nature of observational studies limits our ability to determine causality. We can only adjust for measurable confounders, not unmeasurable ones. Therefore, larger clinical studies should be conducted at higher levels of evidence to validate our findings.

## Conclusion

Our study examined the variability of orbital bony structures among patients with orbital fractures. We demonstrated the symmetry of bilateral orbits and found that patients with ION entry points close to the orbital rim were more likely to sustain IWF after orbital trauma. In contrast, patients with type 1 ION were less likely to sustain IWF. In the future, we intend to construct predictive models that can be validated prospectively. Further, we need a larger sample size and a larger population in the future to improve the accuracy of the test due to the variability of orbital bony structures in different regions and ethnic groups. It would therefore be more appropriate to conduct a multicenter prospective randomized controlled clinical study with a larger sample size.

## Data Availability

Authors will make the raw data supporting conclusions in this article available without undue restriction. Anyone who wishes to obtain the data may contact the corresponding author, Bingjie Shi, shibingjie666@163.com.

## Abbreviations

IWF: Inferior wall fractures
MWF: Medial wall fractures
ION: Infraorbital nerve
IOF: Infraorbital foramen
CT: Computed tomography
HU: Huntsfield units
